# Herbal medicine evaluation for reimbursement-based post-stroke sequelae (HERB-PSS): a retrospective analysis using Korean health insurance claim data, 2020–2024

**DOI:** 10.3389/fneur.2026.1735511

**Published:** 2026-04-20

**Authors:** Soo-Dam Kim, Man Young Park, Jiyun Cha, Changsop Yang, Eunbyul Cho, Sungha Kim

**Affiliations:** 1 KM Science Research Division, Korea Institute of Oriental Medicine, Daejeon, Republic of Korea; 2 Digital Clinical Research Department, Korea Institute of Oriental Medicine, Daejeon, Republic of Korea; 3 Department of Diagnostics, College of Korean Medicine, Wonkwang University, Iksan-si, Republic of Korea

**Keywords:** herbal medicine, Korean medicine, post-stroke sequelae, real-world data, reimbursement, stroke rehabilitation

## Abstract

**Introduction:**

Stroke is a leading cause of long-term disability, and many patients experience persistent neurological and functional impairments after the acute phase. The Korean government launched a national pilot reimbursement program for herbal decoctions to improve access to Korean Medicine (KM) rehabilitation and generate real-world evidence on its safety and effectiveness. This study aimed to analyze the utilization patterns, clinical outcomes, and safety of herbal medicine (HM) among patients with post-stroke sequelae participating in the pilot program.

**Methods:**

A retrospective analysis was conducted using nationwide health insurance claims data from the Health Insurance Review and Assessment Service (HIRA). Patients diagnosed with sequelae of cerebrovascular disease (KCD codes I69 or U234) who received reimbursed HM prescriptions between November 2020 and April 2024 were included. Demographic characteristics, prescription patterns, and symptom severity were analyzed. Symptom changes between the first and last visits were assessed using the Wilcoxon signed-rank test, and adverse events (AEs) were identified from newly added diagnostic codes after HM treatment.

**Results:**

A total of 942 eligible patients were analyzed, with a mean age of 73.2 ± 6.0 years. The majority (57%) initiated KM treatment more than 1 year after stroke onset. The most frequently prescribed formulas were Gamidaebo-tang (23.1%), Mangeum-tang (5.8%), and Gagampalmi-hwan (5.1%). Among 609 patients with paired symptom records, 19.4% showed improvement, 71.9% remained stable, and 8.7% worsened (*p* < 0.001). AEs occurred in 1.03% (10/962) of patients, predominantly gastrointestinal symptoms such as diarrhea (55.6%). All reported AEs were mild and self-limiting.

**Conclusion:**

This study provides the first nationwide real-world evidence on the use, effectiveness, and safety of HM for post-stroke sequelae under Korea’s pilot reimbursement program. Individualized herbal decoctions were widely prescribed and generally well tolerated, showing meaningful symptom stabilization in chronic stroke care. These findings support the feasibility and clinical safety of reimbursed HM and highlight the need for prospective studies to evaluate long-term outcomes, safety, and cost-effectiveness under the expanded second-phase program.

## 1 Introduction

Stroke is a major neurological disorder and one of the leading causes of disability worldwide. Each year, approximately 12 million people experience a new stroke, with ischemic stroke accounting for nearly 65% and hemorrhagic stroke comprising the remainder (1, 2). Advances in acute care have improved survival, but 50–70% of survivors still experience long-term sequelae such as hemiparesis, speech impairment, dysphagia, and cognitive decline, and about 30% remain functionally dependent 6 months after onset (3–5). In South Korea, the age-standardized incidence of stroke declined from 157.8 per 100,000 person-years in 2011 to 118.4 per 100,000 in 2020, while mortality rates steadily decreased due to improvements in acute interventions (6). Nevertheless, up to 70% of survivors report persistent functional deficits, highlighting the substantial burden of post-stroke sequelae on patients and healthcare systems (7, 8). These complications markedly reduce quality of life and increase the demand for rehabilitation services (9).

A Korean nationwide cohort study showed that more than 35% of patients remained dependent in daily activities at 6 months after stroke onset, and nearly half continued to experience persistent functional sequelae (10). Such impairments contribute to significant economic costs, and according to the Korea Disease Control and Prevention Agency, total healthcare expenditures for circulatory diseases in 2022 reached 12.7 trillion KRW, making it the most costly category among chronic diseases (11). Despite the widespread use of antiplatelet agents, anticoagulants, and structured neurorehabilitation programs, functional recovery is often incomplete, particularly in elderly patients or those with severe initial deficits (12–14). Conventional rehabilitation approaches are effective, but they have limitations in restoring long-term independence, and concerns regarding accessibility, cost, and adherence remain (15). Consequently, there is increasing interest in integrative approaches, including complementary therapies, to enhance recovery and mitigate the long-term burden of stroke sequelae (16).

Korean Medicine (KM) has been increasingly utilized for stroke rehabilitation in South Korea. According to national inpatient statistics for 2024, 12,406 patients were admitted to KM hospitals with cerebral infarction and 12,048 with hemiplegia, ranking eighth and ninth among all inpatient diagnoses in KM hospitals (17). Because KM is frequently utilized for the management of post-stroke sequelae and functional impairments during the recovery phase of stroke, these diagnoses likely represent a substantial number of patients receiving KM care for stroke-related sequelae rather than acute stroke management (18). Herbal medicine (HM) is a central component of KM. Several studies have suggested its potential benefits in post-stroke recovery, showing improvements in neurological deficits and mood disorders (19). Experimental research has further demonstrated neuroprotective effects, mediated by anti-inflammatory and anti-apoptotic pathways (20–22). Nevertheless, large-scale real-world studies specifically evaluating HM use for stroke sequelae remain scarce. To address this gap, the Korean government launched a pilot reimbursement program in November 2020 that included HM coverage, thereby creating an unprecedented opportunity to assess utilization and safety using national claims data (23).

The pilot reimbursement program for HM, implemented from November 2020 to April 2024, involved 9,024 KM clinics nationwide and represented the first large-scale national effort to provide public insurance coverage for herbal decoctions. This initiative created a unique opportunity to systematically capture real-world treatment data through the Health Insurance Review and Assessment Service (HIRA) claims, facilitating rigorous evaluation of HM in routine clinical practice. Using these claims data, the present study investigates treatment utilization patterns, patient demographics, adverse event (AE) occurrences, and clinical outcomes among individuals with post-stroke sequelae who received herbal decoctions. The objective is to assess the effectiveness and safety of HM under real-world conditions and to provide evidence regarding its clinical value and policy relevance. Findings from this study are expected to inform healthcare decision-making and support future expansion of insurance coverage for HM in stroke rehabilitation.

## 2 Methods

### 2.1 Study design and objectives

This study adopted a retrospective observational design using national health insurance claims data. It analyzed patients with post-stroke sequelae who received KM treatment reimbursed under the national pilot program for herbal decoctions. The analysis focused on the real-world utilization of herbal prescriptions, patient characteristics, and safety profiles within the policy framework of the national pilot reimbursement program. In addition, exploratory analyses were conducted to examine changes in symptom severity between the first and last recorded visits among patients receiving herbal medicine. Because the study used administrative claims data without a control group, these outcome analyses were intended to describe real-world trends rather than to establish causal treatment effects.

### 2.2 Data source and ethical considerations

All data were obtained from the customized research database of the HIRA^1^ in South Korea. The database contained anonymized claims submitted by licensed KM practitioners participating in the HM reimbursement pilot program. The observation period extended from November 1, 2020, to April 30, 2024, corresponding to the first phase of the national pilot project.

The database was provided through collaboration among the Ministry of Health and Welfare, HIRA, and the National Institute for Korean Medicine Development (NIKOM). Data use was permitted under the program’s research-use policy for policy evaluation and academic analysis. All analyses were performed in a secure remote-access environment to protect personal information. The study protocol was reviewed by the Institutional Review Board of the Korea Institute of Oriental Medicine and was deemed exempt from full review because it involved secondary, de-identified data (Approval No. I-2305/005-003).

### 2.3 Study population and selection criteria

The study population consisted of patients diagnosed with post-stroke sequelae who received reimbursed herbal decoction treatment under the pilot program. Eligible patients were those with valid diagnostic records of sequelae of cerebrovascular disease [Korean Standard Classification of Disease (KCD) codes I69: Sequelae of cerebrovascular disease or U234: Sequelae of stroke] listed as the first secondary diagnosis in the claims database. Because this study used administrative claims data, eligibility was based on diagnostic codes, and imaging or other ancillary clinical data were not available. These diagnostic codes were used to identify patients receiving KM care for stroke-related sequelae during the study period. To ensure diagnostic validity, only individuals whose codes were recorded on or after January 1, 2018, were included. This restriction was applied to ensure that the diagnosis of stroke sequelae was based on relatively recent clinical documentation prior to the implementation of the herbal decoction reimbursement pilot program.

Eligible patients were required to have at least two outpatient visits at participating KM clinics during the pilot period (November 2020–April 2024). Patients were excluded if they had incomplete demographic data, non-stroke diagnoses unrelated to cerebrovascular disease, or records indicating inpatient care in Western medical institutions without subsequent KM follow-up. For safety analyses, all patients who received at least one reimbursed herbal decoction were included. For outcome analyses, only those with symptom severity assessments recorded at least twice were analyzed.

Because many patients sought KM care months or years after the initial cerebrovascular event, visit intervals were highly variable. Follow-up visits were not scheduled at fixed intervals and typically occurred according to patients’ clinical needs. Therefore, each claim record containing herbal prescriptions was treated as an independent treatment episode within the pilot coverage framework rather than a time-fixed course of therapy. This episode-based approach reflects the structure of the pilot reimbursement program, in which herbal decoction prescriptions are recorded and reimbursed on a per-visit basis rather than as a predefined treatment course.

### 2.4 Data pre-processing and variable definitions

The dataset used in this study comprised detailed health insurance claims information, including both billing data and clinical treatment records submitted by KM institutions. Billing information contained claim dates, primary and secondary diagnoses, number of visits, and total treatment days, while treatment records included details on prescribed HM, medical materials used, and other interventions recorded at KM institutions.

Patient demographic characteristics, including age, sex, and comorbidities, were extracted from the claims database. In this study, comorbidities were defined as additional medical conditions recorded as secondary diagnosis codes in the claims database, excluding the primary diagnosis of post-stroke sequelae. Patterns of HM use were examined by identifying commonly prescribed formulas, calculating the cumulative duration of treatment, and analyzing how prescription frequencies varied according to patient characteristics. The cumulative duration of treatment was defined as the total number of days associated with reimbursed herbal decoction prescriptions across all KM visits during the observation period.

For symptom analysis, unstructured text fields describing patients’ chief complaints were processed and standardized. Narrative records were cleaned by removing special characters and normalizing text formats, including spacing, punctuation, and mixed Korean–English expressions. A rule-based keyword categorization approach was then applied to ensure consistent symptom mapping. Stroke-related symptoms were classified into categories such as hemiparesis, sensory disturbance, dizziness, headache, gastrointestinal dysfunction, cognitive impairment, insomnia, dysphagia, urinary disorder, depression, respiratory difficulty, delirium, sleep disorder, and others, as summarized in Supplementary Table 1.

Symptom severity was assessed using a five-point ordinal scale derived from patient self-reports documented in the standardized clinical checklist developed for the pilot reimbursement program. This scale reflects the reported severity of post-stroke sequelae symptoms rather than a comprehensive functional assessment. Severity levels were defined as follows: 1 = minimal symptoms with little impact on daily activities; 2 = mild symptoms with occasional discomfort; 3 = moderate symptoms causing noticeable functional limitation; 4 = severe symptoms substantially interfering with daily activities; and 5 = very severe symptoms requiring continuous clinical attention.

Although standardized functional outcome measures recommended by the StrokEDGE initiative for stroke rehabilitation are widely used in controlled rehabilitation studies (24), the present study relied on the symptom severity scale embedded in the national herbal decoction reimbursement reporting checklist. Because the analysis was based on administrative claims data generated within the policy evaluation framework of the pilot program, this checklist-based measure enabled consistent documentation across participating KM institutions and allowed longitudinal comparison of symptom changes in routine clinical practice.

AEs were identified based on newly added diagnosis codes that appeared after herbal decoction treatment and were classified according to affected organ systems. The treatment phase was defined by the interval between stroke onset and initiation of KM therapy, distinguishing between patients who began treatment within 1 year of stroke onset and those who initiated treatment after 1 year. The total number of KM visits per patient during the program period was also recorded.

### 2.5 Data processing and statistical analysis

All data cleaning and analysis procedures were conducted using R software (version 4.4.1; R Core Team, 2025), following established methodologies for large-scale claims data analyses (25, 26). The dataset was organized into treatment episodes to ensure accurate chronological alignment of each patient’s visits and prescription records. Eligible cases were filtered based on diagnostic codes (KCD code: I69 or U234), treatment settings, and minimum visit requirements.

Descriptive statistical analyses were conducted to summarize demographic characteristics, treatment utilization, and herbal prescription trends. The frequency and total treatment days for HM were analyzed according to sex. These stratified analyses were performed to explore potential demographic differences in treatment utilization and safety outcomes. Follow-up duration was defined as the interval (in days) between the first and last outpatient visits per patient and summarized as mean ± SD to capture variability in treatment continuity.

To evaluate clinical effectiveness, changes in symptom severity between the first and last documented visits were analyzed using the five-point scale defined above.

For effectiveness analyses, only patients with symptom severity scores recorded at both the first and last visits were included. Although all eligible patients had at least two outpatient visits, symptom severity was not consistently documented at every visit in routine clinical practice. Therefore, patients with missing severity data at either time point were excluded from the paired analysis.

Patients were categorized as “Improved” (decrease ≥ 1 point), “Unchanged” (no change), or “Worsened” (increase ≥ 1 point). The Wilcoxon signed-rank test was used to compare ordinal symptom scores before and after treatment.

For safety evaluation, AEs were identified based on newly recorded diagnosis codes appearing after herbal decoction prescriptions. Only diagnosis codes that were not recorded prior to the HM prescription were considered potential adverse events. AE frequencies were summarized as the number and proportion of patients who developed newly recorded diagnosis codes following HM treatment, and the distribution of AEs and associated herbal formulas was examined. Exploratory comparisons were also conducted to examine whether the age distribution of patients experiencing AEs differed between men and women. A fixed lag-time window was not applied because follow-up visits were irregular and not scheduled at standardized intervals in real-world clinical practice.

Inferential statistical analyses were performed using appropriate hypothesis tests. Chi-square or Fisher’s exact tests compared categorical variables such as sex and treatment phase. For continuous variables, independent *t*-tests were used when the data were approximately normally distributed, whereas Wilcoxon rank-sum tests were used when normality assumptions were not satisfied. A two-sided *p*-value <0.05 was considered statistically significant.

## 3 Results

### 3.1 Study population and characteristics

**Figure 1:**
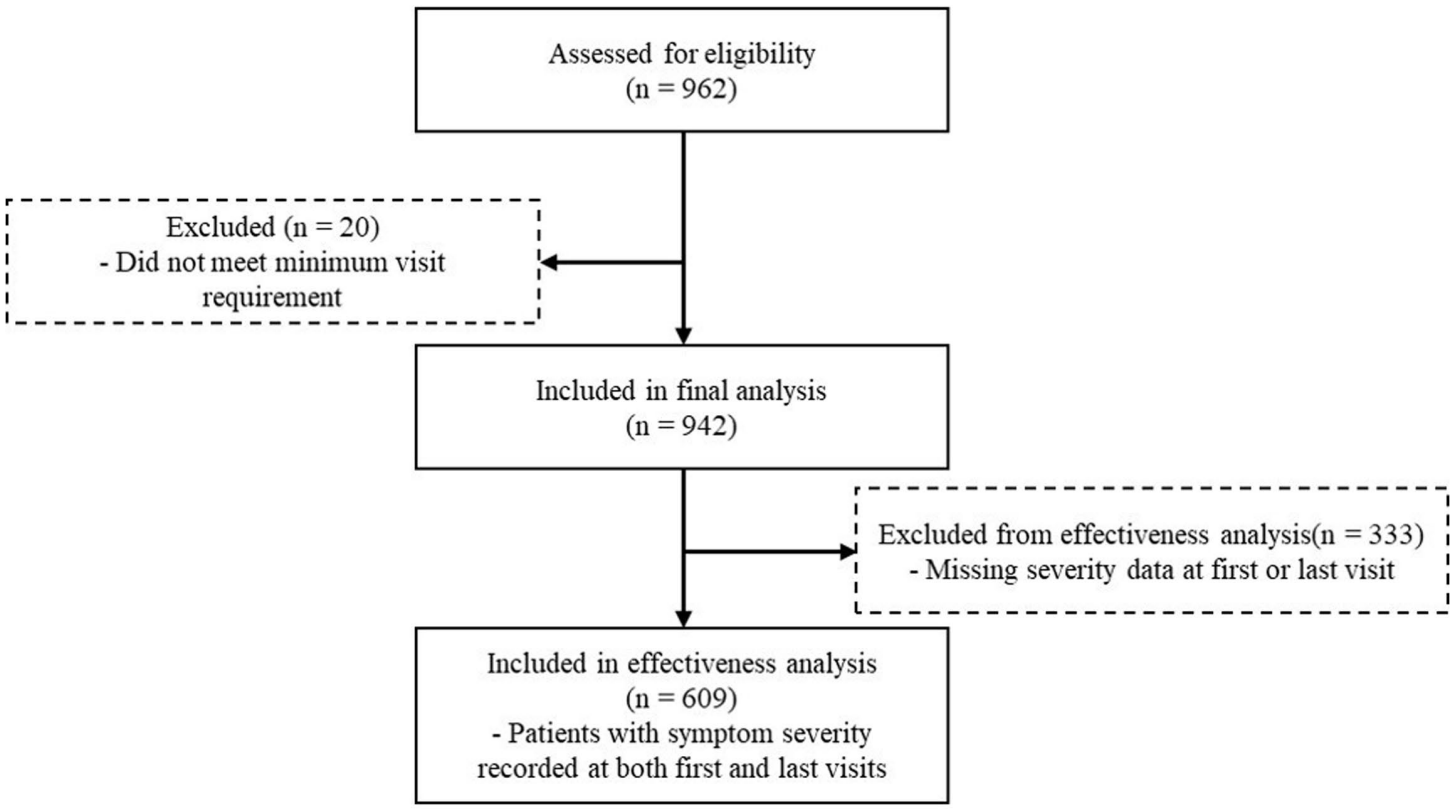
Flow diagram of patient selection and effectiveness analysis.

**Figure 2:**
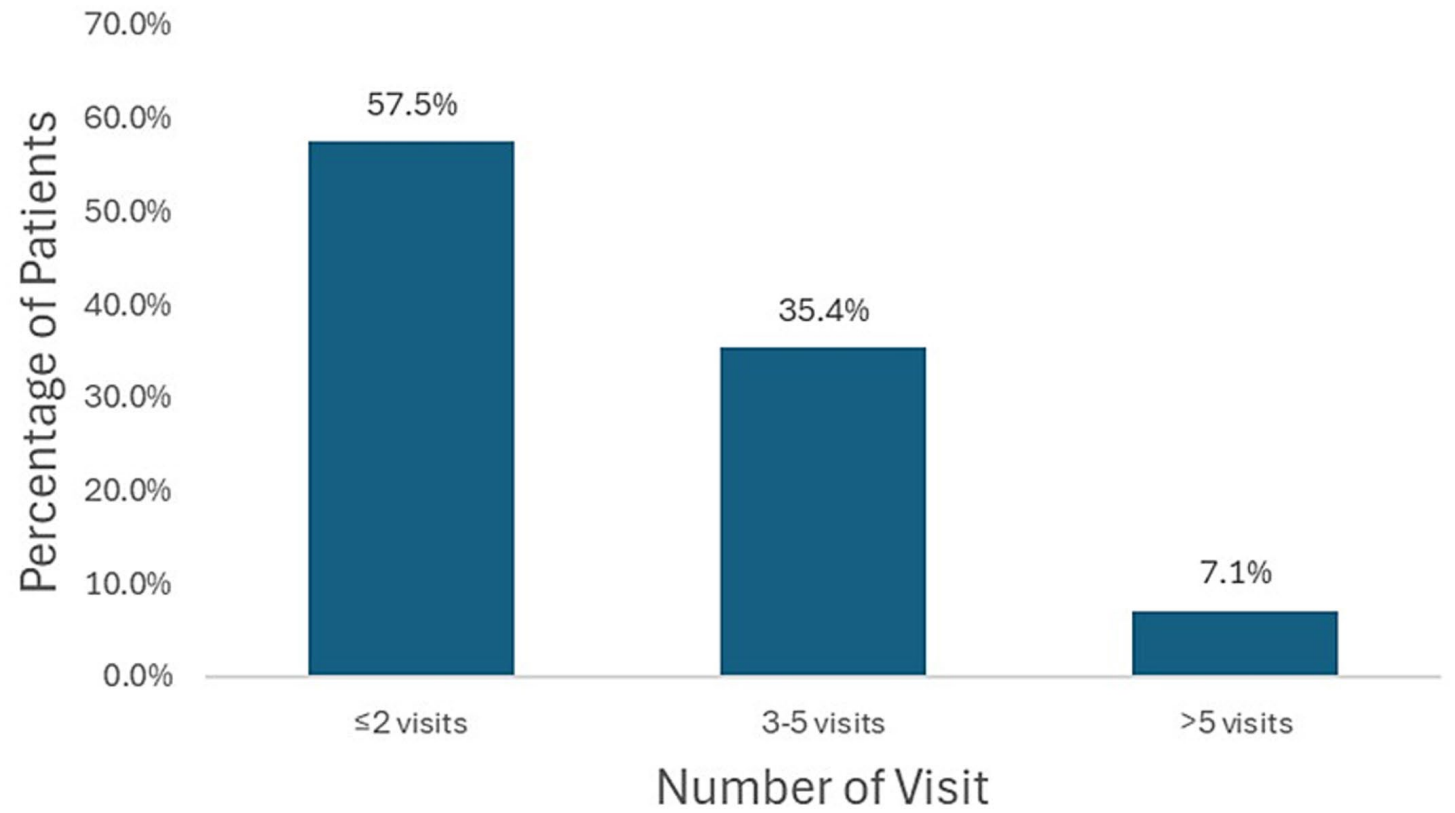
Distribution of patient visits during the study period.

Of the 962 patients diagnosed with sequelae of cerebrovascular disease, a total of 942 individuals who visited KM clinics at least twice were included in the final analysis (Figure 1). Most patients (57.5%) had two or fewer outpatient visits during the observation period, whereas 35.4% visited three to five times, and 7.1% had more than five visits (Figure 2). The mean age of the study population was 73.2 ± 6.0 years, with men being significantly younger than women (72.2 ± 5.9 vs. 74.0 ± 5.9 years, *p* = 0.001). Symptom severity was categorized into five levels. The largest proportion of patients were classified as severity level 3 (40.7%), followed by level 4 (24.2%) and level 2 (21.5%). Levels 1 and 5 accounted for 6.1 and 7.4%, respectively. Male patients showed a slightly higher proportion at level 4 (26.2%) compared with females (22.4%), whereas the overall distribution of severity levels did not differ significantly between sexes (*p* = 0.745). Regarding treatment timing, the mean interval between stroke onset and initiation of KM care was 1,644.7 ± 5,311.5 days overall, 1,752.7 ± 7,441.5 days in men, and 1,554.4 ± 2,354.1 days in women (*p* = 0.596). A total of 405 patients (43.0%) began KM treatment within 1 year of stroke onset, while 537 patients (57.0%) initiated treatment after 1 year. When stratified by sex, there was no significant difference between men and women in the timing of KM treatment initiation (*p* = 0.063) (Table 1).

**Table 1:** Baseline characteristics of study participants.

Variable		Total	Male	Female	*p*-Value
		(*n* = 942)	(*n* = 429)	(*n* = 513)	
Age		73.2 ± 6.0	72.2 ± 5.9	74.0 ± 5.9	<0.001
Symptom severity	1	18 (6.1%)	6 (4.3%)	12 (7.7%)	0.745
	2	64 (21.5%)	31 (22.0%)	33 (21.2%)	
	3	121 (40.7%)	57 (40.4%)	64 (41.0%)	
	4	72 (24.2%)	37 (26.2%)	35 (22.4%)	
	5	22 (7.4%)	10 (7.1%)	12 (7.7%)	
Time from onset to first visit (days), mean ± SD		1644.7 ± 5311.5	1752.7 ± 7441.5	1554.4 ± 2354.1	0.596
Time from onset to first visit (days), IQR		548.0 [98.0;2000.0]	422.0 [93.0;1857.0]	648.0 [103.0;2165.0]	0.163
Within 1 year		405 (43.0%)	199 (46.4%)	206 (40.2%)	0.063
After 1 year		537 (57.0%)	230 (53.6%)	307 (59.8%)	

*p*-Values for continuous variables were derived using independent *t*-tests or Wilcoxon rank-sum tests depending on normality; categorical variables were compared using chi-square tests. SD, standard deviation. Due to skewed distribution, continuous variables are summarized as median [IQR].

### 3.2 Primary symptoms and secondary diagnoses

Primary symptoms of patients with sequelae of cerebrovascular disease were identified through standardized processing of unstructured narrative text fields. Because multiple symptoms could be recorded for a single patient across visits, the counts represent cumulative symptom entries rather than individual patients. Hemiparesis was the most frequently reported symptom, appearing in 1,527 recorded entries (51.1%), followed by unclassified symptoms (11.3%), dizziness or vertigo (9.9%), and sensory disturbance (9.4%). Other reported symptoms included headache (6.4%), gastrointestinal dysfunction (2.5%), and cognitive impairment (1.7%) (Table 2).

**Table 2:** Distribution of recorded primary symptoms.

Primary symptom	*n*	Percent^a^
Hemiparesis	1,527	51.1%
Unclassified symptoms	338	11.3%
Dizziness or vertigo	297	9.9%
Sensory disturbance	280	9.4%
Headache	191	6.4%
Others	156	5.2%
Gastrointestinal dysfunction	75	2.5%
Cognitive impairment	50	1.7%
Insomnia	23	0.8%
Dysphagia	21	0.7%
Depression	16	0.5%
Urinary disorder	9	0.3%
Delirium	4	0.1%
Respiratory difficulty	1	0.0%

a Percentages were calculated using the total number of recorded symptom entries as the denominator because multiple symptoms could be recorded for a single patient across visits.

Regarding secondary diagnoses, the majority of records were associated with cerebrovascular sequelae codes, including I69 (Sequelae of cerebrovascular disease, 51.9%) and U234 (Post-stroke sequelae, 48.0%). A small number of additional diagnoses, such as hemiplegia (G81, 0.0%) and acute nasopharyngitis (J00, 0.0%), were also identified but represented negligible proportions (Table 3).

**Table 3:** Secondary diagnoses distribution.

KCD code	Diagnosis	*n*	Percent
I69	Sequelae of cerebrovascular disease	1,544	51.9
U234	Post-stroke sequelae	1,429	48.0
G81	Hemiplegia	1	0.0
J00	Acute nasopharyngitis (common cold)	1	0.0

KCD, Korean Standard Classification of Disease.

### 3.3 Herbal medicine prescriptions

A total of 91 distinct herbal formulas were prescribed for patients with sequelae of cerebrovascular disease. The most frequently prescribed formula was Gamidaebo-tang (23.1%), followed by Mangeum-tang (5.8%), Gagampalmi-hwan (5.1%), Bojungikki-tang (5.0%), and Hyeongbang-jihwang-tang (4.1%). Other commonly used prescriptions included Geopungjeseup-tang (3.6%), Boyanghwanoh-tang (3.6%), and Sungihwalhyeol-tang (3.3%) (Table 4). The mean duration of herbal decoction prescriptions was 9.98 ± 0.3 days, with no significant difference between men (9.97 ± 0.3 days) and women (9.99 ± 0.3 days, *p* > 0.05).

**Table 4:** Distribution of herbal medicine prescriptions.

No.	Prescription name	*n*	Percent^a^
1	Gamidaebo-tang	668	23.10%
2	Mangeum-tang	168	5.80%
3	Gagampalmi-hwan	148	5.10%
4	Bojungikki-tang	144	5.00%
5	Hyeongbangjihwang-tang	119	4.10%
6	Geopungjeseup-tang	105	3.60%
7	Boyanghwanoh-tang	104	3.60%
8	Sungihwalhyeol-tang	96	3.30%
9	Gagamcheonggan-tang	84	2.90%
10	Guibiondam-tang	80	2.80%
11	Mangeumsopung-tang	68	2.40%
12	Palmul-tang	61	2.10%
13	Ganghwalyupung-tang	58	2.00%
14	Gamiyigigeopung-san	55	1.90%
15	Daeganghwal-tang	54	1.90%
16	Dangguijihwang-tang	52	1.80%
17	Cheongsimyeonja-tang	48	1.70%
18	Sopung-tang	41	1.40%
19	Dodam-tang	28	1.00%
20	Palmulgunja-tang	28	1.00%
21	Gyejigabuja-tang	27	0.90%
22	Oyaksungi-san	27	0.90%
23	Handayeolso-tang	27	0.90%
24	Hyeongbangsabaek-san	25	0.90%
25	Bojungikkitang_Sasang	24	0.80%
26	Yukmihapsaengmaek-san	24	0.80%
27	Galgeunhaegi-tang_Sasang	22	0.80%
28	Yijinsamul-tang	22	0.80%
29	Samguiyangyeong-tang	21	0.70%
30	Ojeok-san	21	0.70%
31	Jeoryeongchajeonja-tang	21	0.70%
32	Gaegyeolseogyeong-tang	20	0.70%
33	Hyangsayangwi-tang	20	0.70%
34	Jowiseungcheong-tang	19	0.70%
35	Bangpungdanggui-san	18	0.60%
36	Yanggyeoksanhwa-tang	18	0.60%
37	Gungshindodam-tang	17	0.60%
38	Yeoldahanso-tang	16	0.60%
39	Banhabaekchulcheonma-tang	15	0.50%
40	Yangyeong-tang	15	0.50%
41	Sopunghwalhyeol-tang	14	0.50%
42	Cheongsimondam-tang	14	0.50%
43	Insamyangyeong-tang	13	0.40%
44	Jihwangeumja	13	0.40%
45	Geopungsokmyeong-tang	12	0.40%
46	Sosihotang	12	0.40%
47	Gyeonjeong-san	11	0.40%
48	Guibi-tang	11	0.40%
49	Cheongsimjihwang-tang	10	0.30%
50	Geopung-tang	9	0.30%
51	Saryuk-tang	9	0.30%
52	Gamicheongsim-tang	8	0.30%
53	Sopungbosimdodam-tang	8	0.30%
54	Jeongjeongamiyijintang	7	0.20%
55	Boyeumhwalhyeol-tang	6	0.20%
56	Cheongsanggeopung-tang	6	0.20%
57	Gwakhyangjeonggi-san_Sasang	5	0.20%
58	Dodamsungi-tang	5	0.20%
59	Sosokmyeong-tang	5	0.20%
60	Sopungsungi-won	5	0.20%
61	Yanghyeolgeopung-tang	5	0.20%
62	Jayumganghwa-tang	5	0.20%
63	Cheongsimyeoldae-tang	5	0.20%
64	Hyeongbangdojeok-san	5	0.20%
65	Hyeongbangpaedok-san_Sasang	5	0.20%
66	Banhasasim-tang	4	0.10%
67	Bangpungtongseong-san	4	0.10%
68	Yeongseonjeitong-eum	4	0.10%
69	Jayun-tang	4	0.10%
70	Ssanghap-tang	3	0.10%
71	Cheonmabanha-tang	3	0.10%
72	Cheongrijagam-tang	3	0.10%
73	Hyangsayukgunja-tang	3	0.10%
74	Gamisoyosan (pattern-based)	2	0.10%
75	Daejingyo-tang	2	0.10%
76	Doksampalmul-tang	2	0.10%
77	Bangpung-tang	2	0.10%
78	Sogeonjung-tang	2	0.10%
79	Sopungcheongsim-tang	2	0.10%
80	Seungyangyikkibuja-tang	2	0.10%
81	Sibimigwanjung-tang	2	0.10%
82	Yukul-tang	2	0.10%
83	Cheonmagudeung-eum	2	0.10%
84	Gunggwichongso-yijung-tang	1	0.00%
85	Daeseunggi-tang	1	0.00%
86	Baekho-tang	1	0.00%
87	Bopaewon-tang	1	0.00%
88	Bulhwangeumdan (decoction)	1	0.00%
89	Bulhwangeumjeonggi-san	1	0.00%
90	Sambi-tang	1	0.00%
91	Hyeolbuchukeo-tang	1	0.00%

a Percentages represent the proportion of each prescription among all recorded herbal medicine prescriptions.

### 3.4 Effectiveness of KM treatment

**Figure 3:**
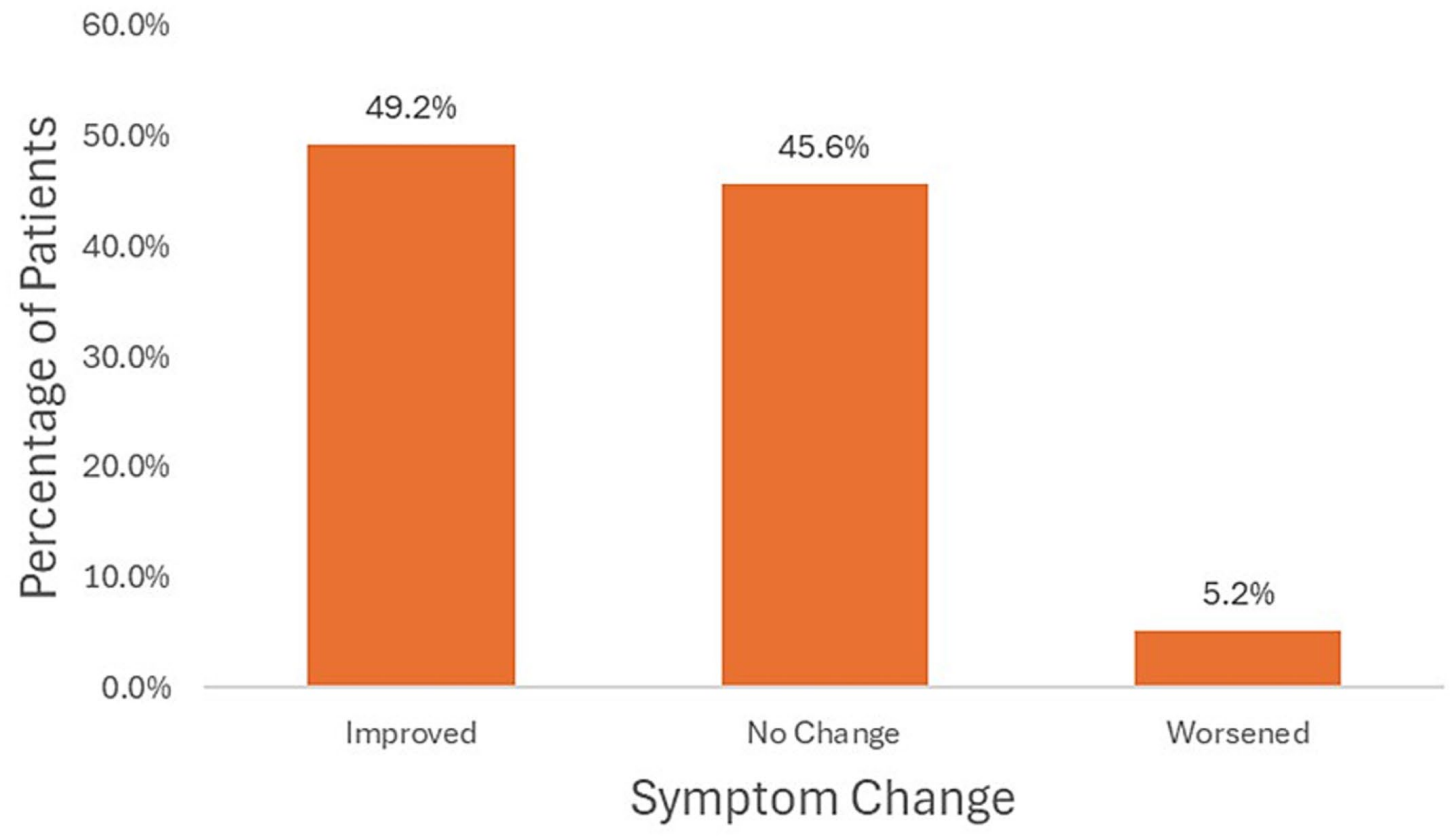
Changes in symptom severity after KM treatment.

Among the 942 eligible patients, a total of 609 individuals who had recorded symptom severity scores at both the first and last visits were included in the effectiveness analysis. At the final visit, 282 patients (46.3%) were classified as severity level 3, followed by 158 (25.9%) at level 2, 103 (16.9%) at level 4, 51 (8.4%) at level 1, and 15 (2.5%) at level 5. Comparison of paired symptom scores using the Wilcoxon signed-rank test indicated a significant overall improvement in symptom severity after KM treatment (*p* < 0.001) (Table 5). Based on the change in symptom scores between visits, 118 patients (19.4%) showed improvement, 438 (71.9%) exhibited no change, and 53 (8.7%) experienced worsening of symptoms (Figure 3).

**Table 5:** Changes in symptom severity between the first and last visits among patients with severity scores available at both time points (*n* = 609).

	Point	Final symptom severity					Total	*p*-value^a^
		1	2	3	4	5		
Initial symptom severity	1	35 (76%)	8 (17%)	3 (7%)	0 (0%)	0 (0%)	46 (8%)	<0.001
	2	14 (10%)	104 (73%)	23 (16%)	2 (1%)	0 (0%)	143 (23%)	
	3	2 (1%)	38 (14%)	215 (80%)	15 (6%)	0 (0%)	270 (44%)	
	4	0 (0%)	7 (6%)	37 (32%)	71 (61%)	2 (2%)	117 (19%)	
	5	0 (0%)	1 (3%)	4 (12%)	15 (45%)	13 (39%)	33 (5%)	
	Total	51 (8%)	158 (26%)	282 (46%)	103 (17%)	15 (2%)	609 (100%)	

a Wilcoxon signed-rank test.

### 3.5 Safety evaluation

Among the 962 patients with post-stroke sequelae, a total of 10 individuals (1.03%) experienced AEs during HM treatment. The most frequently reported AEs were gastrointestinal in nature, including diarrhea (55.6%), constipation (11.1%), and abdominal bloating (11.1%). Other reported symptoms included tongue stiffness (11.1%) and musculoskeletal discomfort in the lower limbs (11.1%) (Table 6). The mean age of patients who experienced AEs was 73.9 ± 3.2 years, with no statistically significant difference between men (74.2 ± 3.3 years) and women (72.5 ± 3.5 years) (*p* = 0.527). The herbal formulas most frequently associated with AEs were Gamidaebo-tang (20.0%) and Galgeunhaegi-tang_sasang (20.0%), followed by Gunggwichongsoyijung-tang (10.0%), Mankeum-tang (10.0%), Samgwiyangyeong-tang (10.0%), Yijinsamul-tang (10.0%), Insamyangyeong-tang (10.0%), and Hyeongbangdojeok-san (10.0%) (Table 7).

**Table 6:** Distribution of reported adverse events.

System	Adverse event symptom	*n*	Percent^a^
Gastrointestinal	Diarrhea	5	55.6%
	Constipation	1	11.1%
Others	Abdominal bloating	1	11.1%
	Tongue stiffness	1	11.1%
	Leg and muscle/joint discomfort	1	11.1%

a Percentages were calculated using the total number of reported adverse events as the denominator.

**Table 7:** Adverse event incidence by herbal formula.

Prescription name	AE cases	Percent	AE rate^a^
Gamidaebo-tang	2	20.0%	0.30%
Galgeunhaegi-tang_sasang	2	20.0%	9.09%
Gunggwichongsoyijung-tang	1	10.0%	100.00%
Mankeum-tang	1	10.0%	0.60%
Samgwiyangyeong-tang	1	10.0%	4.76%
Yijinsamul-tang	1	10.0%	0.55%
Insamyangyeong-tang	1	10.0%	7.69%
Hyeongbangdojeok-san	1	10.0%	20.00%

a AE rate represents the proportion of adverse events relative to the total number of prescriptions for each herbal formula. AE, adverse event.

## 4 Discussion

This study is the first to comprehensively analyze the utilization patterns, effectiveness, and safety of HM for post-stroke sequelae within the Korean national pilot reimbursement program using large-scale health insurance claims data. The findings provide real-world evidence on how herbal decoctions are being prescribed and utilized for stroke rehabilitation and demonstrate the clinical and policy relevance of integrating KM into the national health insurance system. Several East Asian countries have adopted different models to institutionalize traditional medicine within public healthcare, with Japan emphasizing standardized Kampo formulations under physician-based prescriptions and China promoting broader access to traditional medical services through hospital-based integration and provincial insurance schemes (27, 28). The Korean pilot program is unique in targeting individualized herbal decoctions prescribed according to clinical pattern differentiation. By focusing on specific disease indications and leveraging real-world claims data, this program offers a pragmatic model for data-driven policy evaluation and integration of traditional medicine within a modern reimbursement framework.

A total of 942 patients with sequelae of cerebrovascular disease were analyzed, with most receiving short-term herbal prescriptions averaging about 10 days, consistent with the reimbursement limit defined in the first-phase pilot program (23). Because different analyses required specific data elements, the analytic sample size varied across outcomes, reflecting the structure of routinely collected claims data. The mean age of participants was 73.2 years, notably higher than that reported in other KM studies for chronic diseases, reflecting the older population commonly affected by stroke (29, 30). Although there were no significant sex-based differences in treatment initiation timing or symptom severity, a considerable proportion of patients (57%) began KM treatment more than 1 year after stroke onset. This delayed initiation suggests that many patients turned to KM as a complementary option after conventional treatment or during chronic stages when functional recovery may have plateaued. Previous studies have reported that stroke survivors often seek complementary or integrative therapies when they are dissatisfied with the outcomes of conventional treatment or when complications or persistent symptoms arise during the course of recovery (31, 32). In contrast, early rehabilitation interventions have been shown to improve neurological outcomes, highlighting the importance of timely access to KM treatment in the early phase of post-stroke care (33).

The most commonly prescribed formulas were Gamidaebo-tang (23.1%), Mangeum-tang (5.8%), and Bojungikki-tang (5.0%), reflecting clinical strategies traditionally applied for neurological and functional recovery in post-stroke patients (34–36). Other frequently used prescriptions included Gagampalmi-hwan (5.1%), Hyeongbangjihwang-tang (4.1%), Geopungjeseup-tang (3.6%), and Boyanghwanoh-tang (3.6%), indicating a diverse therapeutic landscape. These formulas share therapeutic principles such as reinforcing qi and blood circulation and tonifying deficiency, addressing the chronic deficiency and stagnation patterns common in post-stroke patients. Their effects are thought to involve multiple biological mechanisms, including anti-inflammatory, antioxidative, and anti-apoptotic actions, modulation of the blood–brain barrier, and promotion of neurogenesis and angiogenesis, which together facilitate neural repair and functional recovery after stroke (37). These effects are partly mediated through key signaling pathways such as PI3K/Akt, which regulate neuroinflammation, oxidative stress, and apoptosis following ischemic injury (38). The diversity of prescriptions highlights the individualized nature of KM, where treatment is tailored to each patient’s constitution and symptoms rather than standardized protocols. This variability reflects the inherent flexibility of KM practice, consistent with previous findings on the adaptability of herbal therapy in complex chronic conditions (39, 40).

In terms of treatment effectiveness, 19.4% of patients showed improvement in symptom severity, while 71.9% remained stable and 8.7% worsened. The Wilcoxon signed-rank test confirmed a statistically significant overall improvement after KM treatment (*p* < 0.001). Although the improvement rate may seem modest compared with clinical trial settings (41), it should be interpreted within the real-world context where many patients initiated KM therapy months or even years after stroke onset, when neuroplastic recovery potential is naturally reduced. However, because this study was based on observational claims data without a control group, the observed symptom stability cannot be interpreted as a causal treatment effect. Symptom stability may also reflect the natural course of recovery or the influence of concurrent rehabilitation and other unmeasured factors. Furthermore, considering evidence that rehabilitation combined with HM interventions can improve outcomes, integrating integrative care into post-stroke rehabilitation may further enhance recovery and quality of life (19, 42).

Safety analysis showed a low incidence of AEs (1.03%, 10 out of 962 patients), with gastrointestinal symptoms such as diarrhea (55.6%) and abdominal bloating (11.1%) being the most frequently reported, followed by constipation (11.1%). These adverse reactions were consistent with those previously reported in HM research, confirming the overall safety of KM treatment under real-world clinical conditions (43). The overall AE rate was comparable to or even lower than those observed in previous large-scale studies on the safety of HM, supporting the favorable safety profile of reimbursed herbal decoctions (44, 45). The herbal formulas most commonly associated with AEs were Gamidaebo-tang (20.0%) and GalgeunHaegi-tang_Sasang (20.0%), but their absolute incidence was minimal and did not raise specific safety concerns. Overall, AEs were mild and self-limiting, reflecting the good tolerability of herbal decoction therapy in patients with post-stroke sequelae. Continued pharmacovigilance remains essential as the scale of herbal reimbursement continues to expand.

The major strength of this study lies in its use of nationwide, real-world data from the HIRA, enabling a comprehensive evaluation of KM utilization patterns among patients with cerebrovascular sequelae. Unlike previous small-scale or single-institution studies, this large claims-based dataset reflects actual clinical practice across thousands of KM institutions, thereby providing robust and generalizable evidence on the real-world applicability of HM within Korea’s healthcare system. These findings support the feasibility and safety of reimbursed herbal decoctions for chronic neurological rehabilitation and suggest that the integration of KM into post-stroke care pathways may contribute to reducing long-term disability and improving patients’ quality of life.

Several limitations should be acknowledged. First, as a retrospective analysis of claims data, detailed clinical information such as imaging findings, standardized neurological assessments, and comprehensive rehabilitation histories was unavailable. The five-point symptom severity scale used in this study, although appropriate for large-scale analysis, has not been externally validated against established stroke outcome measures. In addition, because symptom severity was recorded only when available in the reimbursement reporting checklist, analyses requiring repeated severity assessments were limited to a subset of patients with both baseline and follow-up records available. Second, potential underreporting or misclassification of AEs may have occurred because only coded entries were analyzed, while irregular follow-up intervals, the absence of a fixed lag-time window, and unaccounted comorbid conditions may have further limited the detection and attribution of adverse events. Third, the observational design inherently limits causal inference regarding the effects of HM on symptom improvement or AE occurrence. Unmeasured confounding factors such as concurrent rehabilitation, medication adherence, and socioeconomic status may also have influenced the observed outcomes. Future studies with predefined follow-up schedules are warranted to better evaluate the temporal effects of treatment. In addition, prospective cohort or pragmatic trial designs incorporating validated tools such as the Modified Rankin Scale (mRS) or NIH Stroke Scale (NIHSS) to confirm and extend these findings (46–48).

The first-phase pilot program (2020–2024) provided a critical foundation for evaluating the clinical feasibility and policy impact of reimbursed herbal decoction therapy in South Korea. By including conditions such as post-stroke sequelae within the coverage scope, the initiative reduced patients’ financial burden and improved accessibility to KM rehabilitation services. Building upon this foundation, the second-phase pilot program (2024–2026) has expanded participation to 7,805 KM and general medical institutions nationwide and doubled the reimbursed treatment period from 10 to 20 days per condition annually (49). This expansion presents an opportunity to generate broader, more representative evidence regarding the cost-effectiveness, safety, and long-term clinical outcomes of HM under real-world conditions. Future studies should leverage this extended framework to explore treatment utilization trends, dose–response relationships, and institutional variations in outcomes. Such analyses will be essential for developing optimized, evidence-based reimbursement strategies and for advancing the integration of KM into comprehensive stroke rehabilitation pathways.

## 5 Conclusion

This study provides real-world evidence on the utilization, effectiveness, and safety of HM for post-stroke sequelae within Korea’s national pilot reimbursement program. Analysis of large-scale health insurance claims showed that individualized herbal decoctions were widely prescribed and generally well tolerated, with a very low incidence of AEs. These findings indicate that HM may play a supportive role in symptom stabilization and long-term functional maintenance in chronic stroke rehabilitation. However, the lack of detailed clinical data limits causal inference. Future prospective studies under the expanded second-phase program should assess long-term outcomes, safety, and cost-effectiveness to inform evidence-based and sustainable policy development.

## Data Availability

The data analyzed in this study is subject to the following licenses/restrictions: data supporting the findings of this study are available upon reasonable request to the corresponding author. Requests to access these datasets should be directed to Sungha Kim, bozzol@kiom.re.kr.

## Footnotes

1
https://www.hira.or.kr
