# Supplementary material for: Herbal medicine evaluation for reimbursement-based post-stroke sequelae (HERB-PSS): a retrospective analysis using Korean health insurance claim data, 2020–2024
Source: Front Neurol. 2026 Apr 20;17:1735511. doi: 10.3389/fneur.2026.1735511 (PMC13137878; doi:10.3389/fneur.2026.1735511)
Supplement: Supplementary file 1 [file Table_1.DOCX]

**Supplement 1.** Symptom Classification

| Category | Included Symptoms | Key Terms |
| --- | --- | --- |
| 1. Hemiparesis | Facial paralysis | “paralysis”, “Bell’s palsy”, “facial palsy”, “Guanwasa”, “orbicularis oculi”, “numbness” |
|  | Peripheral facial palsy |  |
|  | Bell’s palsy |  |
|  | Muscle weakness |  |
| 2. Sensory Disturbance | Sensory dullness | “reduced sensation”, “sensory disturbance”, “tingling”, “numbness”, “hypesthesia” |
|  | Tingling sensation |  |
|  | Hypesthesia |  |
|  | Paresthesia |  |
| 3. Dizziness | Vertigo | “dizziness”, “vertigo”, “lightheadedness” |
|  | Dizziness |  |
|  | Light-headedness |  |
| 4. Headache | Migraine | “headache”, “migraine”, “occipital pain”, “posterior headache”, “temporal pain” |
|  | Occipital headache |  |
|  | Posterior headache |  |
|  | Temporal pain |  |
| 5. Gastrointestinal | Nausea | “digestion”, “nausea”, “vomiting”, “difficulty swallowing” |
|  | Vomiting |  |
|  | Dysphagia |  |
| 6. Dysfunction | Memory decline | “cognition”, “memory”, “concentration”, “judgment” |
|  | Poor concentration |  |
|  | Reduced judgment |  |
| 7. Insomnia | Insomnia | “insomnia”, “sleep disorder”, “trouble sleeping” |
|  | Sleep disturbance |  |
|  | Difficulty initiating sleep |  |
| 8. Dysphagia | Difficulty swallowing | “dysphagia”, “difficulty swallowing”, “swallowing disorder” |
|  | Swallowing difficulty |  |
|  | Swallowing disorder |  |
| 9. Urinary Disorder | Difficulty urinating | “urination”, “urine”, “bladder” |
|  | Frequent urination |  |
|  | Urinary incontinence |  |
| 10. Depression | Depressive mood | “depression”, “anxiety”, “fatigue”, “loss of motivation” |
|  | Loss of motivation |  |
|  | Anxiety |  |
| 11. Respiratory Difficulty | Shortness of breath | “breathing”, “shortness of breath”, “dyspnea” |
|  | Dyspnea |  |
|  | Breathing discomfort |  |
| 12. Delirium | Confusion | “delirium”, “confusion”, “mental disorder”, “altered consciousness” |
|  | Mental disturbance |  |
|  | Consciousness disorder |  |
| 13. Sleep Disorder | Hypersomnia | “sleep disorder”, “hypersomnia”, “insomnia”, “sleep rhythm disturbance” |
|  | Insomnia |  |
|  | Disturbed sleep rhythm / Sleep rhythm disturbance |  |
| 14. Others | Convulsion | “spasm”, “edema”, “pain”, “fatigue” |
|  | Edema |  |
|  | Pain |  |
|  | Fatigue |  |
